# 8-Ammonio­naphthalene-2-sulfonate monohydrate: the zwitterionic hydrate of 1,7-Cleve’s acid

**DOI:** 10.1107/S1600536809030396

**Published:** 2009-08-08

**Authors:** Graham Smith, Urs D. Wermuth, David J. Young

**Affiliations:** a School of Physical and Chemical Sciences, Queensland University of Technology, GPO Box 2434, Brisbane 4001, Australia; b School of Biomolecular and Physical Sciences, Griffith University, Nathan, Qld, 4111, Australia

## Abstract

The structure of 8-amino-2-naphthalene­sulfonic acid monohydrate (1,7-Cleve’s acid hydrate), C_10_H_9_NO_3_S·H_2_O, shows the presence of a sulfonate–aminium group zwitterion, both groups and the water mol­ecule of solvation giving cyclic *R*_3_^3^(8) O—H⋯O and N—H⋯O inter­molecular hydrogen-bonding inter­actions, forming chains which extend down the *a* axis of the unit cell. Additional peripheral associations, including weak aromatic ring π–π inter­actions [centroid–centroid distance = 3.6299 (15) Å], result in a two-dimensional sheet structure.

## Related literature

1,7-Cleve’s acid and 1,6-Cleve’s acid have important industrialchemical applications as azo dye precursors, see: O’Neil (2001 ▶). For the preliminary crystal data for a number of aminona­phthalenesulfonic acids, see: Corbridge *et al.* (1966 ▶). For the strutures of 5-amino-2-naphthalene­sulfonic acid (1,6-Cleve’s acid) and the 1:1 adduct of 1,7-Cleve’s acid with strychnine, see: Smith *et al.* (2004 ▶, 2007 ▶).
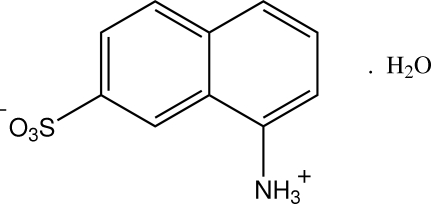


## Experimental

### 

#### Crystal data

C_10_H_9_NO_3_S·H_2_O
*M* *_r_* = 241.27
Orthorhombic, 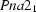

*a* = 7.1616 (3) Å
*b* = 16.4608 (7) Å
*c* = 8.9059 (3) Å
*V* = 1049.88 (7) Å^3^
*Z* = 4
Mo *K*α radiation
μ = 0.31 mm^−1^
*T* = 297 K
0.35 × 0.20 × 0.05 mm

#### Data collection

Oxford Diffraction Gemini-S CCD-detector diffractometer
Absorption correction: multi-scan (*SADABS*; Sheldrick, 1996 ▶) *T*_min_ = 0.950, *T*_max_ = 0.990
5904 measured reflections
2252 independent reflections
1830 reflections with *I* > 2σ(*I*)
*R*_int_ = 0.034

#### Refinement

*R*[*F* ^2^ > 2σ(*F* ^2^)] = 0.036
*wR*(*F* ^2^) = 0.076
*S* = 0.91
2252 reflections
165 parameters
1 restraint
H atoms treated by a mixture of independent and constrained refinement
Δρ_max_ = 0.29 e Å^−3^
Δρ_min_ = −0.17 e Å^−3^
Absolute structure: Flack (1983 ▶), 974 Friedel pairs
Flack parameter: 0.06 (8)

### 

Data collection: *CrysAlis CCD* (Oxford Diffraction, 2008 ▶); cell refinement: *CrysAlis RED* (Oxford Diffraction, 2008 ▶); data reduction: *CrysAlis RED*; program(s) used to solve structure: *SIR92* (Altomare *et al.*, 1994 ▶); program(s) used to refine structure: *SHELXL97* (Sheldrick, 2008 ▶); molecular graphics: *PLATON* (Spek, 2009 ▶); software used to prepare material for publication: *PLATON*.

## Supplementary Material

Crystal structure: contains datablocks I, global. DOI: 10.1107/S1600536809030396/tk2500sup1.cif

Structure factors: contains datablocks I. DOI: 10.1107/S1600536809030396/tk2500Isup2.hkl

Additional supplementary materials:  crystallographic information; 3D view; checkCIF report

## Figures and Tables

**Table 1:** Hydrogen-bond geometry (Å, °)

*D*—H⋯*A*	*D*—H	H⋯*A*	*D*⋯*A*	*D*—H⋯*A*
O1*W*—H11*W*⋯O23^i^	0.82 (4)	1.98 (4)	2.798 (3)	176 (3)
O1*W*—H12*W*⋯O22^ii^	0.90 (3)	1.91 (3)	2.796 (3)	169 (3)
N8—H81⋯O23^iii^	0.88 (3)	1.95 (3)	2.817 (2)	171 (2)
N8—H82⋯O21^ii^	0.89 (4)	1.96 (3)	2.793 (3)	154 (3)
N8—H83⋯O1*W*	0.98 (4)	1.82 (4)	2.725 (3)	152 (4)

Symmetry codes: (i) 
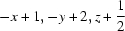
; (ii) 
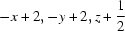
; (iii) 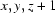.

**Fig. 1.**
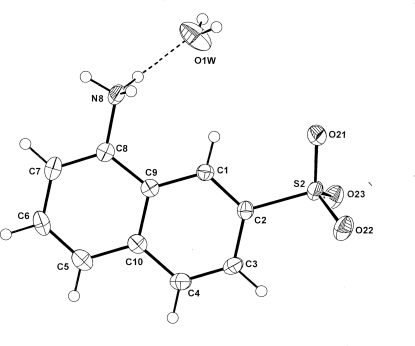
Molecular configuration and atom naming scheme for the 1,7-Cleve's acid zwitterion and the water molecules in (I). Displacement ellipsoids are drawn at the 50% probability level. The inter-species hydrogen bond is shown as a dashed line.

**Fig. 2.**
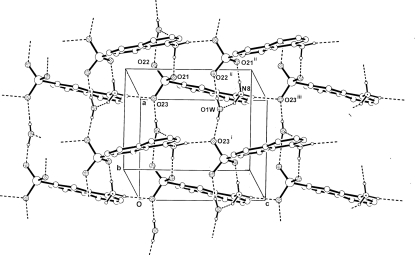
The 2-D hydrogen-bonded sheet structure of (I) Viewed down the approximate b axial direction of the unit cell showing also the intermolecular cyclic R33(8) sulfonate–aminium–water association. Non-interactive hydrogen atoms are omitted and hydrogen-bonds are shown as dashed lines.

**Fig. 3.**
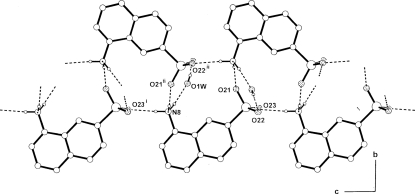
The sheet structure of (I) viewed down the b axial direction; hydrogen-bonds are shown as dashed lines.

## supplementary crystallographic information

### Comment

8-Amino-2-naphthalenesulfonic acid (1-naphthylamine-7-sulfonic acid: 1,7-Cleve's acid) is a compound which along with 1,6-Cleve's acid has important industrial chemical applications as an azo dye precursor (O'Neil, 2001). Although the preliminary crystal data for a number of aminonaphthalenesulfonic acids were reported by Corbridge *et al.* (1966), the crystal structures of very few have been determined. We reported the structure of 5-amino-2-naphthalenesulfonic acid (1,6-Cleve's acid) (Smith *et al.*, 2004) which, together with the 1:1 adduct of 1,7-Cleve's acid with strychnine (Smith *et al.*, 2007) represent the only crystallographically characterized examples. In both of these structures the molecules exist as sulfonate–amino group zwitterions as is commonly the case with the aminosulfonic acids.

The crystals used for the determination of the structure reported here, the hydrate C_10_H_9_NO_3_S.H_2_O (I), were obtained from the attempted preparation of a co-crystal with picrylsulfonic acid in ethanol-water solvent, the usual reported form of the acid is the same monohydrate. The unit-cell parameters and space group reported for this compound (Corbridge *et al.*, 1964) (orthorhombic, *a* = 8.91, *b* = 16.44, *c* = 7.14 Å, space group *P*2_1_*cn* (non-standard setting) are comparable to those determined here for (I).

The molecules of 1,7-Cleve's acid monohydrate in (I), like those of the isomeric anhydrous 1,6-Cleve's acid (Smith *et al.*, 2004), not unexpectedly show the presence of a sulfonate–aminium group zwitterion (Fig. 1). However, the presence of the water molecule of solvation in (I) results in significantly different hydrogen-bonding characteristics. Whereas with 1,6-Cleve's acid, the aminium protons give interactions with the sulfonate-O acceptors of three separate acid species, giving a 3-D structure, in (I) the structure is 2-D (Fig. 2). The primary intermolecular sulfonate–aminium group interaction involves the water molecule in a cyclic *R*_3_^3^(8) association resulting in chains extending down the *a* direction in the unit cell. Additional peripheral interactions (Table 1) together with weak intermolecular π–π aromatic ring associations [minimum ring centroid separation, 3.6299 (15) Å for the six-membered ring C5–C10], give the structure extension across *c* (Fig. 3).

### Experimental

The title compound (I) was isolated as the only product from the attempted preparation of an adduct compound of 8-amino-2-naphthalenesulfonic acid (1-naphthylamine-7-sulfonic acid) with picrylsulfonic acid, by heating together for 10 min under reflux 1 mmol quantities of the two reagents in 40 ml of 50% ethanol-water. The crystals formed as colourless flat prisms after partial room-temperature evaporation of the hot-filtered solution.

### Refinement

Hydrogen atoms potentially involved in hydrogen-bonding interactions were located by difference methods and their positional and isotropic displacement parameters were refined. Other H atoms were included in the refinement at calculated positions (C–H = 0.93 Å) as riding models with *U*_iso_ fixed at 1.2*U*_eq_(C).

### Crystal data

**Table:**

C_10_H_9_NO_3_S·H_2_O	*F*(000) = 504
*M**_r_* = 241.27	*D*_x_ = 1.526 Mg m^−^^3^
Orthorhombic, *P**n**a*2_1_	Mo *K*α radiation, λ = 0.71073 Å
Hall symbol: P 2c -2n	Cell parameters from 2826 reflections
*a* = 7.1616 (3) Å	θ = 3.1–32.3°
*b* = 16.4608 (7) Å	µ = 0.31 mm^−^^1^
*c* = 8.9059 (3) Å	*T* = 297 K
*V* = 1049.88 (7) Å^3^	Flat prism, colourless
*Z* = 4	0.35 × 0.20 × 0.05 mm

### Data collection

**Table:**

Oxford Diffraction Gemini-S CCD-detector diffractometer	2252 independent reflections
Radiation source: Enhance (Mo) X-ray tube	1830 reflections with *I* > 2σ(*I*)
graphite	*R*_int_ = 0.034
ω scans	θ_max_ = 27.5°, θ_min_ = 3.1°
Absorption correction: multi-scan (*SADABS*; Sheldrick, 1996)	*h* = −9→9
*T*_min_ = 0.950, *T*_max_ = 0.990	*k* = −21→21
5904 measured reflections	*l* = −11→11

### Refinement

**Table:**

Refinement on *F*^2^	Secondary atom site location: difference Fourier map
Least-squares matrix: full	Hydrogen site location: inferred from neighbouring sites
*R*[*F*^2^ > 2σ(*F*^2^)] = 0.036	H atoms treated by a mixture of independent and constrained refinement
*wR*(*F*^2^) = 0.076	*w* = 1/[σ^2^(*F*_o_^2^) + (0.0476*P*)^2^] where *P* = (*F*_o_^2^ + 2*F*_c_^2^)/3
*S* = 0.91	(Δ/σ)_max_ = 0.003
2252 reflections	Δρ_max_ = 0.29 e Å^−^^3^
165 parameters	Δρ_min_ = −0.17 e Å^−^^3^
1 restraint	Absolute structure: Flack (1983), 974 Friedel pairs
Primary atom site location: structure-invariant direct methods	Flack parameter: 0.06 (8)

### Special details

**Table:**

Geometry. Bond distances, angles *etc*. have been calculated using the rounded fractional coordinates. All su's are estimated from the variances of the (full) variance-covariance matrix. The cell e.s.d.'s are taken into account in the estimation of distances, angles and torsion angles
Refinement. Refinement of *F*^2^ against ALL reflections. The weighted *R*-factor *wR* and goodness of fit *S* are based on *F*^2^, conventional *R*-factors *R* are based on *F*, with *F* set to zero for negative *F*^2^. The threshold expression of *F*^2^ > σ(*F*^2^) is used only for calculating *R*-factors(gt) *etc*. and is not relevant to the choice of reflections for refinement. *R*-factors based on *F*^2^ are statistically about twice as large as those based on *F*, and *R*- factors based on ALL data will be even larger.

### Fractional atomic coordinates and isotropic or equivalent isotropic displacement parameters (Å^2^)

**Table:**

	*x*	*y*	*z*	*U*_iso_*/*U*_eq_	
S2	0.91078 (7)	0.91246 (3)	0.30596 (6)	0.0279 (2)	
O21	0.9227 (3)	0.98981 (10)	0.38255 (19)	0.0426 (6)	
O22	1.0718 (2)	0.89287 (11)	0.21446 (19)	0.0432 (6)	
O23	0.7375 (3)	0.90447 (11)	0.21899 (17)	0.0386 (6)	
N8	0.7911 (4)	0.89568 (12)	0.9061 (2)	0.0316 (7)	
C1	0.8622 (3)	0.85751 (14)	0.5919 (3)	0.0244 (6)	
C2	0.8963 (3)	0.83679 (13)	0.4461 (2)	0.0247 (6)	
C3	0.9158 (4)	0.75485 (14)	0.4020 (3)	0.0313 (7)	
C4	0.8930 (4)	0.69545 (14)	0.5046 (3)	0.0328 (7)	
C5	0.8196 (4)	0.65100 (14)	0.7627 (3)	0.0350 (7)	
C6	0.7800 (4)	0.66940 (15)	0.9072 (3)	0.0377 (8)	
C7	0.7701 (4)	0.75056 (16)	0.9537 (2)	0.0344 (8)	
C8	0.8000 (3)	0.81129 (15)	0.8546 (2)	0.0266 (7)	
C9	0.8386 (3)	0.79617 (14)	0.7015 (2)	0.0244 (6)	
C10	0.8515 (3)	0.71317 (14)	0.6564 (3)	0.0270 (7)	
O1W	0.6039 (4)	1.01561 (13)	0.7583 (3)	0.0636 (9)	
H1	0.85450	0.91190	0.61930	0.0290*	
H3	0.94410	0.74190	0.30290	0.0380*	
H4	0.90490	0.64150	0.47490	0.0390*	
H5	0.82590	0.59690	0.73300	0.0420*	
H6	0.75920	0.62790	0.97610	0.0450*	
H7	0.74290	0.76270	1.05330	0.0410*	
H81	0.762 (4)	0.8959 (14)	1.002 (3)	0.038 (6)*	
H82	0.904 (5)	0.9182 (18)	0.897 (4)	0.051 (9)*	
H83	0.690 (7)	0.927 (3)	0.860 (4)	0.056 (10)*	
H11W	0.502 (6)	1.0367 (18)	0.746 (4)	0.076 (10)*	
H12W	0.699 (4)	1.0497 (17)	0.738 (3)	0.082 (8)*	

### Atomic displacement parameters (Å^2^)

**Table:**

	*U*^11^	*U*^22^	*U*^33^	*U*^12^	*U*^13^	*U*^23^
S2	0.0338 (3)	0.0303 (3)	0.0196 (2)	−0.0020 (2)	0.0031 (3)	0.0018 (2)
O21	0.0683 (14)	0.0271 (8)	0.0325 (9)	−0.0069 (9)	0.0053 (10)	0.0014 (7)
O22	0.0422 (11)	0.0546 (11)	0.0327 (9)	−0.0010 (9)	0.0135 (9)	0.0033 (8)
O23	0.0390 (10)	0.0510 (11)	0.0258 (8)	0.0022 (9)	−0.0032 (8)	0.0010 (7)
N8	0.0405 (14)	0.0348 (12)	0.0195 (10)	−0.0027 (10)	0.0037 (10)	−0.0013 (8)
C1	0.0254 (12)	0.0231 (10)	0.0247 (10)	0.0015 (9)	−0.0001 (9)	−0.0007 (8)
C2	0.0233 (11)	0.0272 (10)	0.0236 (10)	−0.0016 (10)	0.0026 (9)	0.0027 (8)
C3	0.0366 (13)	0.0313 (11)	0.0260 (10)	0.0014 (11)	0.0054 (11)	−0.0064 (9)
C4	0.0370 (13)	0.0266 (11)	0.0347 (12)	0.0026 (11)	0.0025 (12)	−0.0049 (9)
C5	0.0330 (12)	0.0277 (12)	0.0442 (14)	0.0006 (11)	0.0001 (11)	0.0054 (11)
C6	0.0385 (15)	0.0351 (13)	0.0395 (14)	0.0001 (12)	0.0002 (13)	0.0163 (12)
C7	0.0338 (14)	0.0455 (14)	0.0239 (11)	−0.0030 (12)	0.0025 (11)	0.0048 (11)
C8	0.0230 (12)	0.0308 (12)	0.0259 (11)	−0.0014 (10)	−0.0005 (9)	0.0002 (9)
C9	0.0211 (11)	0.0263 (11)	0.0259 (10)	0.0000 (9)	−0.0009 (9)	−0.0002 (9)
C10	0.0217 (12)	0.0270 (12)	0.0323 (11)	0.0002 (10)	0.0005 (10)	0.0019 (10)
O1W	0.0410 (12)	0.0407 (12)	0.109 (2)	0.0022 (11)	−0.0058 (14)	0.0170 (12)

### Geometric parameters (Å, °)

**Table:**

S2—O21	1.4470 (17)	C4—C10	1.415 (4)
S2—O22	1.4484 (16)	C5—C10	1.413 (4)
S2—O23	1.469 (2)	C5—C6	1.352 (4)
S2—C2	1.766 (2)	C6—C7	1.401 (4)
O1W—H12W	0.90 (3)	C7—C8	1.351 (3)
O1W—H11W	0.82 (4)	C8—C9	1.413 (3)
N8—C8	1.464 (3)	C9—C10	1.427 (3)
N8—H81	0.88 (3)	C1—H1	0.9300
N8—H82	0.89 (4)	C3—H3	0.9300
N8—H83	0.98 (4)	C4—H4	0.9300
C1—C2	1.365 (3)	C5—H5	0.9300
C1—C9	1.415 (3)	C6—H6	0.9300
C2—C3	1.412 (3)	C7—H7	0.9300
C3—C4	1.348 (4)		
			
O21—S2—O22	114.47 (11)	N8—C8—C9	118.54 (19)
O21—S2—O23	112.16 (11)	C7—C8—C9	122.1 (2)
O21—S2—C2	106.91 (10)	N8—C8—C7	119.38 (18)
O22—S2—O23	110.86 (10)	C1—C9—C10	118.78 (19)
O22—S2—C2	106.70 (10)	C8—C9—C10	116.9 (2)
O23—S2—C2	105.06 (10)	C1—C9—C8	124.3 (2)
H11W—O1W—H12W	113 (3)	C5—C10—C9	119.6 (2)
H81—N8—H83	103 (3)	C4—C10—C9	118.7 (2)
H82—N8—H83	114 (2)	C4—C10—C5	121.7 (2)
C8—N8—H81	108.6 (15)	C2—C1—H1	120.00
C8—N8—H82	109 (2)	C9—C1—H1	120.00
H81—N8—H82	108 (3)	C4—C3—H3	120.00
C8—N8—H83	111 (3)	C2—C3—H3	120.00
C2—C1—C9	120.0 (2)	C3—C4—H4	119.00
S2—C2—C3	118.12 (16)	C10—C4—H4	119.00
S2—C2—C1	120.44 (17)	C10—C5—H5	120.00
C1—C2—C3	121.4 (2)	C6—C5—H5	120.00
C2—C3—C4	119.5 (2)	C5—C6—H6	120.00
C3—C4—C10	121.6 (2)	C7—C6—H6	120.00
C6—C5—C10	120.6 (2)	C6—C7—H7	120.00
C5—C6—C7	120.4 (2)	C8—C7—H7	120.00
C6—C7—C8	120.3 (2)		
			
O21—S2—C2—C1	12.5 (2)	C10—C5—C6—C7	0.0 (4)
O21—S2—C2—C3	−169.2 (2)	C6—C5—C10—C4	−180.0 (3)
O22—S2—C2—C1	135.40 (18)	C6—C5—C10—C9	−1.1 (4)
O22—S2—C2—C3	−46.3 (2)	C5—C6—C7—C8	0.0 (4)
O23—S2—C2—C1	−106.85 (19)	C6—C7—C8—N8	−179.3 (2)
O23—S2—C2—C3	71.4 (2)	C6—C7—C8—C9	1.2 (4)
C9—C1—C2—S2	175.82 (16)	N8—C8—C9—C1	−2.7 (3)
C9—C1—C2—C3	−2.4 (3)	N8—C8—C9—C10	178.2 (2)
C2—C1—C9—C8	−179.0 (2)	C7—C8—C9—C1	176.9 (2)
C2—C1—C9—C10	0.1 (3)	C7—C8—C9—C10	−2.2 (3)
S2—C2—C3—C4	−175.6 (2)	C1—C9—C10—C4	1.9 (3)
C1—C2—C3—C4	2.7 (4)	C1—C9—C10—C5	−177.0 (2)
C2—C3—C4—C10	−0.6 (4)	C8—C9—C10—C4	−179.0 (2)
C3—C4—C10—C5	177.2 (3)	C8—C9—C10—C5	2.1 (3)
C3—C4—C10—C9	−1.7 (4)		

### Hydrogen-bond geometry (Å, °)

**Table:**

*D*—H···*A*	*D*—H	H···*A*	*D*···*A*	*D*—H···*A*
O1W—H11W···O23^i^	0.82 (4)	1.98 (4)	2.798 (3)	176 (3)
O1W—H12W···O22^ii^	0.90 (3)	1.91 (3)	2.796 (3)	169 (3)
N8—H81···O23^iii^	0.88 (3)	1.95 (3)	2.817 (2)	171 (2)
N8—H82···O21^ii^	0.89 (4)	1.96 (3)	2.793 (3)	154 (3)
N8—H83···O1W	0.98 (4)	1.82 (4)	2.725 (3)	152 (4)
C1—H1···O21	0.93	2.52	2.899 (3)	105
C1—H1···N8	0.93	2.61	2.913 (3)	100
C6—H6···O22^iv^	0.93	2.53	3.281 (3)	137

Symmetry codes: (i) −*x*+1, −*y*+2, *z*+1/2; (ii) −*x*+2, −*y*+2, *z*+1/2; (iii) *x*, *y*, *z*+1; (iv) *x*−1/2, −*y*+3/2, *z*+1.
